# The effect of Non- ionizing electromagnetic field with a frequency of 50 Hz in Rat ovary: A transmission electron microscopy study

**Published:** 2016-02

**Authors:** Amir Afshin Khaki, Arash Khaki, Seyed Shahin Ahmadi

**Affiliations:** 1 *Tabriz Health Services Management Research Center, Tabriz University of Medical Sciences, Tabriz, Iran. *; 2 *Department of Pathobiology, Tabriz Branch, Islamic Azad University, Tabriz, Iran.*

**Keywords:** *Electromagnetic field*, *Ovary*, *Organelles*, *Rat*

## Abstract

**Background:**

Recently, there are increasing concerns and interests about the potential effects of Electromagnetic Field (EMF) on both human and animal health.

**Objective:**

The goal of this study was to evaluate the harmful effects of 50 Hz non-ionizing EMF on rat oocytes.

**Materials and Methods:**

In this experimental study 30 rats were randomly taken from laboratory animals and their ags and weights were determined. These 3 month's old rats were randomly divided into 3 groups. The control group consisted of 10 rats without receiving any treatment and kept under normal conditions. Experimental group 1 (10 rats) received EMF for 8 weeks (3 weeks intrauterine +5 weeks after births) and experimental group 2 (10 rats) received EMF for 13 weeks (3 weeks intrauterine +10 weeks after birth). After removing the ovaries and isolating follicles, granulosa cells were fixed in glutaraldehyde and osmium tetroxide. Electron microscopy was used to investigate the traumatic effects of EMF on follicles.

**Results:**

In control group nucleus membrane and mitochondria in follicle’s cytoplasm seemed normal in appearance. Theca layer of primary follicles in experimental group was separated clearly, zona layer demonstrated trot with irregular thickness and ovarian stroma seemed isolated with dilated vessels showing infiltration.

**Conclusion:**

According to the results of this study, it can be concluded that EMF has harmful effects on the ovarian follicles.

## Introduction

In recent years, attention to the effects of Extremely Low Frequency Electromagnetic Fields (ELF- EMF) and Radio Frequency Electromagnetic Fields (RF-EMF) on safety of environment and health has increased (1). The non-ionizing radiation fields with its low frequency, long wavelength and low leverage do not have enough energy to release an electron from its orbit. With the increasing usage of electricity in daily life, humans are more than ever are exposed to ELF-EMF (2). ELF-EMF has a frequency range of 1 to 100 Hz that is released from variety of sources such as power lines, electric transport systems, and home appliances (3). 

While high-frequency fields with sufficient energy could cause cancer, the risks of ELF-EMF on human health are still controversial (2, 4). Studies have shown that ELF-EM fields can cause brain tumors, childhood leukemia, genotoxicity, neurodegenerative diseases, congenital malformations, miscarriage and infertility (5, 6). "Teratogenic", is the name given to factors that impair the development of the embryo or fetus during pregnancy. Overall, teratogens include environmental factors and factors related to the mother (7). 

In recent decades, extensive studies to determine the effects of traumatic ELF-EMF fields on the reproductive system and development of the fetus in animal and humans models have been conducted (8-10). EM fields potentially affect fetal development, but the mechanism of effect is still not completely understood. Development of tissues, organs and systems during embryogenesis is sensitive to toxic agents. Based on studies, exposure to ELF-EMF can increase the side effects of birth (birth defect). 

Studies on the effects of 50-100 Hz electromagnetic fields in embryos of different species of animals (fish, chicken, rat and mouse) shows that the early stages of embryonic development is sensitive to the electromagnetic field radiation. TV and mobile phone use during the first trimester of pregnancy increase the risk of fetal growth restriction, especially in women with a history of high-risk pregnancy (11, 12). Exposure to ELF-EMF during pregnancy can cause adverse effects on pregnancy and fetal development in female rats. Also reduction in the speed up of fetal weight in late pregnancy and abnormalities in the fetus is observed (13). 

EMF prevents the formation and differentiation of neural stem cells during embryonic development (14). ELF-EMF has significant effect on basic neural functions and synaptic plasticity (15). Increasing number of skeletal abnormalities in rat embryos affected by 50 Hz electromagnetic field is also reported (16). Environmental radiation may affect embryonic development, the immune system and lungs. A new epidemiological evidence of increased risk of asthma in offspring of mothers exposed to EMF during pregnancy is presented (17). The results of the studies showed a possible link between maternal occupational exposure with ELF-EMF and brain tumors in children (4). 

An increased risk of leukemia in children whose mothers exposed to high levels of occupational exposure with ELF-EMF during pregnancy, have been reported (5). While other studies, did not report any complications on fertility and embryo development of mice and humans exposed to EMF (14). Based on studies of exposure to ELF-EMF between the 1^st^-20^th^ day of pregnancy and organogenesis period there is no biological effect on the fetus (7). It has also been shown that exposure of mice to EMF during the organogenesis period is not significantly teratogenic (7). 

Based on empirical evidence of the adverse effects of EMF on embryonic development of amphibians and birds, the effects of these fields on mammals still needs to be studied. Due to vulnerability of the fetus to toxic agents and often contradictory reports, the aim of this study was to investigate the biological effects of EMF on reproduction, fertility and embryonic development.

## Materials and methods

In this experimental study rats were taken randomly from laboratory animals and their ages and weight were determined. Three months old Wistar rats 300±30 gr were selected for the study. Animals were then randomly divided to three groups; Control group (n=10), Experiment 1 (E1) (n=10) and Experiment 2 (E_2_) (n=10). During the study, three groups were maintained and fed in the same condition. Experimental groups were exposed to 50-60 Hz magnetic field. Group E1 (10 rats) received EMF for 8 weeks (including 3 weeks intrauterine +5 weeks after births) and group E_2_ (10 rats) received EMF for 13 weeks (including 3 weeks intrauterine +10 weeks after birth). After the above mentioned periods of proestrus cycles, rats with pentobarbital (40 mg) were scarified under anesthesia. 

The study design was based on the morphological structure of the follicles and secondary follicles were as primary follicles seen and grafian follicles were. After removal of the ovaries and isolating the follicles, follicles cells were placed in fixative and required samples were prepared by electron microscopy. Samples of electron microscope were fixed with glutaraldehyde solution to prevent deformation of cell structure. Samples were taken from the three groups for electron microscope, put into glutaraldehyde and then osmium tetroxide 2% for fixation. After processing the examined samples were embedded in resin. Semi-thin sections were stained with Toluidine blue and thin sections stained with lead citrate and uranyl acetate and eventually examined with Leo906 electron microscope.

In the present study, an EMF generator was used to produce a 50 Hz magnetic field. The EMF generating device was made based on the theory of Helmholtz. The Helmholtz coils have a diameter of 20 cm and contain 200 windings of 0.8 mm copper wire located at a distance of 10 cm from each other. The devices used in this study were: an AC power supply with a voltage of 0-250 volts, a two- channel oscilloscope to check the parameters and waveforms of the windings input and output, a multimeter to measure the current voltage, AC and DC teslameter with an accuracy of 0.001 mT, a thermometer with an accuracy of 0.1^o^C, and an incubator. In the selection of these devices, parameters such as the need to achieve a uniform field with certain intensity were carefully considered. Fennel seeds were purchased from local markets and authenticated by a botanist (School of Pharmacy, Tabriz University of Medical Sciences, Tabriz, Iran). 

The extract was prepared according to the World Health Organization (WHO) protocol for preparation of an alcoholic extract (7). Briefly, 100 g of fruit was shed-dried, powdered, and added to 1000 ml of 70% ethanol (v/v) and left to macerate at room temperature for 20 hr. The basin was slowly rotated during this time. After filtration, ethanol evaporated at low pressure at 30^o^C. Ovarian tissue samples with a clean surface were transferred on a plate containing washing solution (pH=7.4) and washed several times to remove clots and tissue debris, so the blood stains, clot adhesion, and debris were cleaned. Then, the samples were divided into 5 mm sections. The samples, after being placed in a solution of 2.5% glutaraldehyde, were washed with 0.1 M phosphate buffer solution (pH=7.4) for 6 hr. Subsequently, they were kept for 2 hr in 1% osmium tetroxide solution. Later, they were washed 3 times with 0.1 M phosphate-buffered saline (pH=7.4). 

For hydration, alcohol (ethanol) was used with increasing concentration gradient. Replacement was performed using propylene oxide. Samples’ molding procedure was performed by Epon 812 resin. Trimmed samples were installed on the ultra-microtome (Reichert- Jung, Germany). Semi-thin sections with 500-700 nanometers thickness and a speed of 2.5 mm per second were prepared. They were stained with toluidine blue solution. After preparing the ultra-thin sections, in order to stain the grids, uranyl acetate solution 3% and lead citrate were used for 1-2 hr. This stage of the research was conducted at the Research Center of Tabriz University of Medical Sciences. 

## Results

**Figure 1.**
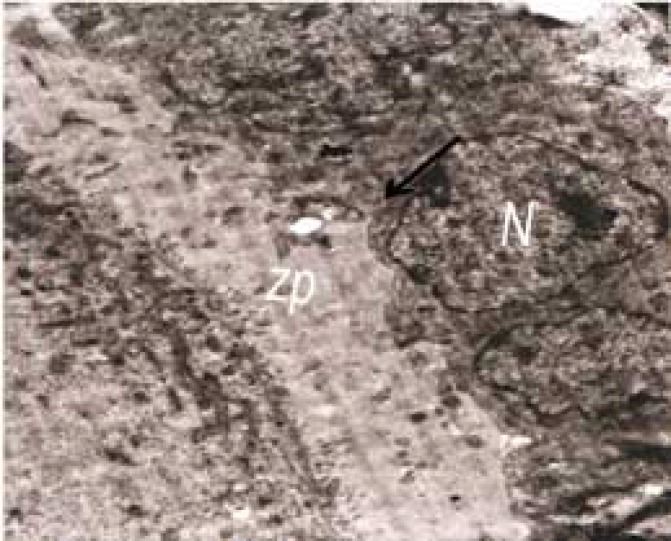
Ovary of control group 1: Electron micrograph of microvilli (MI) and cytoplasmic projection visible in the zona pellucida (zp) (32000×).

Control group showed cytoplasmic projection visible in zona pellucida, normal nucleus membrane beside round and oval shaped mitochondria (Figure 1). The figures in experimental groups showed theca cells having nuclei in different stages of heterochromatinization, a sign of degeneration and vacuolization was obvious in electron micrographs as a sign of cell degeneration. 

**Figure 2:**
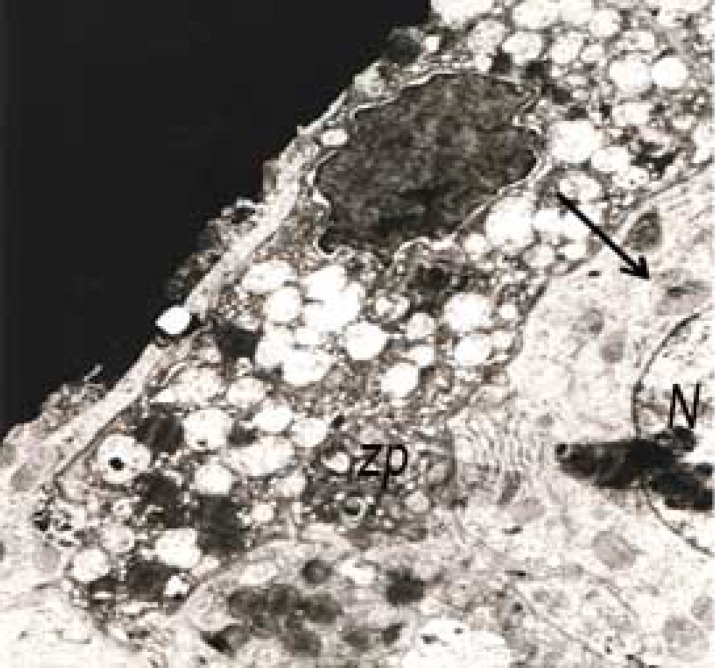
Ovary in experimental group 1: Electron micrograph showed theca cells with nuclei in different stages of heterochromatinization, a sign of degeneration. Vacuolization is obvious (arrow) (8000×).

**Figure 3:**
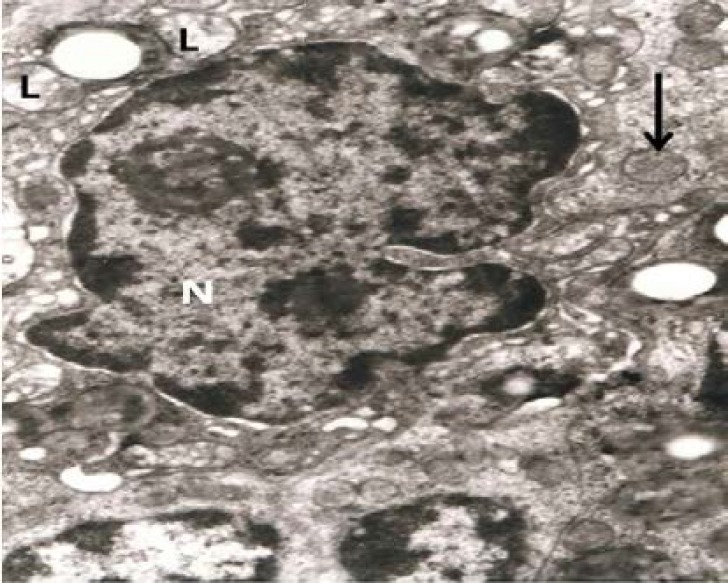
Ovary Experimental Group 1-2: Follicular cell with a highly indented and heterochromatic nucleus (N) with indistinct nuclear membrane seen in the photomicrograph. Mitochondrial cristae are decreased (arrow head). Lipid droplets and vacuolization is clearly visible (arrow) (33000 X

In addition, follicular cell with a highly indented and heterochromatic nucleus and indistinct nuclear membrane was seen in the electron micrograph. Mitochondrial cristae were decreased and lipid droplets and vacuolization was clearly visible in electron micrograph (Figure 2, 3). Nucleus were heterochromatic and swollen. 

**Figure 4:**
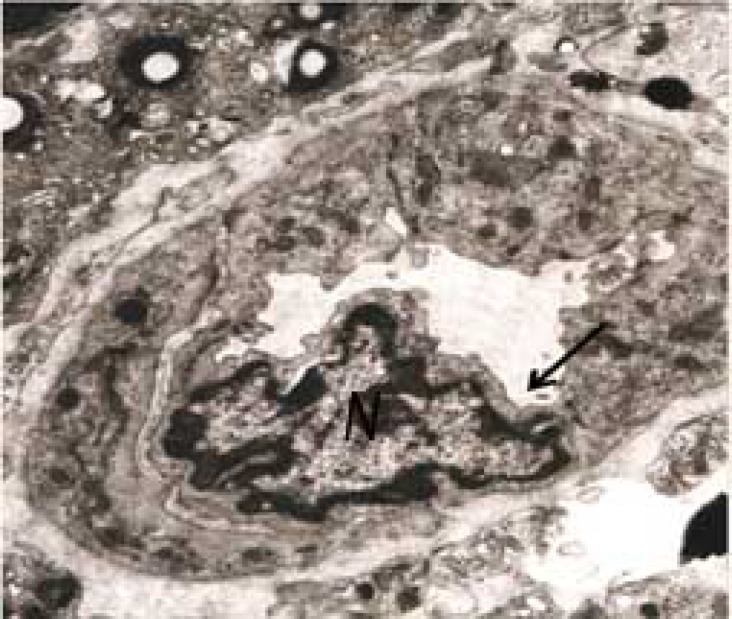
Ovaryin experimental group 2: Electron micrograph with precapillary prominent endothelial cells. Nucleus is heterochromatic and swollen (arrow). Mitochondria, golgi apparatus, rough endoplasmic reticulum (rER) and free ribosomes are seen in the cytoplasm (20000×).

**Figure 5:**
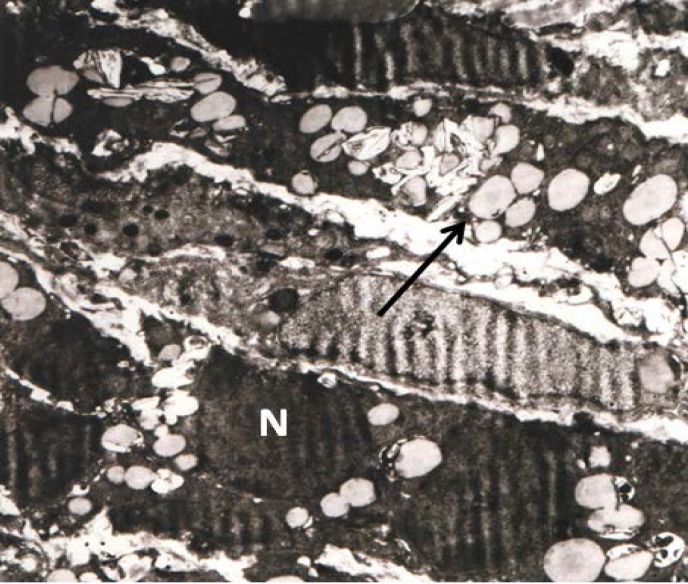
Ovary in experimental group 2: Electronmicrograph of a cell from granulosa layer with an irregular dark nucleus (N) with several intracytoplasmic empty vacuoles and dark granules. The vacuoles have a ‘soap sud’ appearance (arrow) (17000×).

Mitochondria, golgi apparatus, rough Endoplasmic Reticulum (rER) and free ribosomes were seen in the cytoplasm, granulose cells layer showed an irregular dark nucleus (N) with several intracytoplasmic empty vacuoles and dark granules. The vacuoles had a ‘soap sud’ appearance (Figures 4, 5).

## Discussion

Although many studies on animals have tried to investigate the effects of electromagnetic waves on reproductive, the results were controversial. Many studies suggest that exposure to EMF can have complications in reproduction and embryo development and a link is sugessted between excessive exposure to electromagnetic waves and abortion (18, 19). 

Fertility in female mammals is limited with the number of lost eggs before birth (20, 21). Ovarian follicles consist of an oocyte and granulosa cells around it that are essential for the survival and maturation of eggs. According to a study of TEM early signs of degeneration of the nuclear membrane as infolding were observed. Initial morphological changes in granulosa cells as granulosa cells’ retraction, loss of microvilli and the lack or condensing of mitochondrial cristae, occur at the same time with the start of apoptosis in granulosa cells and can show the cytotoxic effects of EMF (22). 

Growing primary follicles with relatively irregular arrangement and interrupted layers of theca in some parts of the follicles and zona pellucida with heterogeneous thickness were visible. Ovarian stroma in terms of construction, dilated blood vessels and blood congestion was obvious. Also, infiltration of white blood cells was visible. Shrinkage of oocyte nucleoli and the irregular shape of the oocytes were observed in this study. Maturation, fertilization and embryonic development before implantation (pre- implantation embryo development) depend on oocyte growth and differentiation of follicular cells around it (21). Irregular morphology of the core can be a sign of changes in nuclear structure (23). A study conducted by Han *et al* on the effects of EMF on ovarian follicles showed that oocyte nucleus becomes smaller and changes shape (15). Figure 6 shows that zona pellucida in follicles were highly degraded and not visible. Also, WBC aggregations for local or topical infiltration were seen around the follicles. 

Based on the results, exposure to extremely low frequency magnetic fields may impair fertility of female mammals by reducing the ability of follicles to reach the stage of development which is an essential prerequisite for successful reproductive .It has been shown that microvilli of the oocyte and granulosa cells that contact through gap junction were involved in nutrition of oocytes (23). It was found that the very ELF-EMF reduced the DNA synthesis of granulosa cells in the follicle and by reducing the ability of follicles to achieve high level of development, cause harmful effects on the fertility in female mammals (reproductive system) (23-25). 

The studies about the effects of EMF on ovarian follicles have shown that oocyte nucleus becomes smaller and changes shape and the number of apoptotic bodies and autophagic vacuoles in granulosa cells increased, compared to the control group (26, 27). According to a study conducted by Roshangar *et al* it was found that exposure to EMF can lead to morphological changes in the oocyte and increasing the nuclear congestion of granulosa cells (28). EMF exposure in daily life reduces the supply of egg cell in the ovaries and will lead to an increase in infertility (7, 25, 26). It showed that follicle atresia in the ovaries is associated with apoptosis of granulosa cells. Programmed death (apoptosis) of granulosa cells in the presence of EMF has been reported (23).

The EMF guideline gives an overview of the current knowledge regarding EMF-related health risks and provides concepts for the diagnosis, treatment and accessibility measures of NHS to improve and restore individual health outcomes as well as for the development of strategies for prevention (29). This study found a significant decrease in the number of ovarian follicles in rats exposed to EMF (24). Other studies did not report any damaging effects of exposure on reproduction or fertility in rats and humans. Studies employing real mobile phone exposures demonstrate an almost 100% consistency in showing adverse effects (30). 

No significant differences were found in the body weight and weight of ovaries between exposed and sham exposed rats. The mean number of primordial follicles in the ovaries were significantly lower in exposed rats as compared to sham exposed rats. The authors conclude that an abdominal exposure of rats to a 900 MHz electromagnetic field could reduce the number of primordial follicles in the ovaries (24). Our results showed that follicles had pyknotic nucleus with condensed cytoplasm. Cortex of ovary had only a few follicles. Signs of degeneration and cystic changes were seen. Follicular cell had pyknotic nucleus. Many spaces were created due to edema. Light microscopic studies showed that zona pellucida had uneven thickness. Electron microscopic study of zona pellucida showed that microvilli of oocyte and cytoplasmic projections (filopodia) of the follicular cells in experimental group 1 were lost. 

The control group showed these cytoplasmic processes in an array. This finding was important because of their role in the nutrition of oocyte; with the diminution in their number, the capacity of nutrient absorption is compromised. This study suggested that loss of this function of the zona pellucida aggravated the oocyte degeneration. Under LM nuclei of follicular and theca interna cells showed pyknotic features. Spaces were seen between the cells due to edema. Follicular cell under EM showed irregular nucleus with several intracytoplasmic vacuoles and dark granules. The nuclei of the dying granulosa and the theca interna were dark because of loss of all major organelles that perform biological activities. 

Granulosa cells had few dilated mitochondria and endoplasmic reticulum, while the other organelles were less affected. It showed decrease in synthesizing activity of protein and lipid, as another sign of cell degeneration. As a defense mechanism, vacuolization occurred in the follicular and theca cells as a demand to sequester lipid material. This is done by enzymes within the vacuoles that the cells create. Free radicals can hamper the cellular physiology resulting in the formation of cytotoxic materials which are stored within such vacuoles. Theca layer showed abundant tissue debris between the cells giving it "onion ring" and lamellar appearance. The remains of tissue debris were seen after cytoplasmic destruction. Coordinated sequence of events in the ovarian follicle results in production of a mature egg. It involves various cell types such as the oocyte and the surrounding granulosa and theca cells. Metarteriole/pre-capillaries showed endothelial swelling and infolding of nuclear membrane with peripheral concentration of chromatin clumps, resulting in decrease in size of the lumen in experimental group 1. This can account for the edema seen in this group.

## Conclusion

According to the results of this study, it can be concluded that EMF has harmful effects on oocyte and the ovarian follicles. In general, these findings indicated that exposure to EMF had a deleterious effect on gonadal tissue and gametogenesis, which were observed in ovary and were more pronounced in the former. Thus it can be concluded that long term exposure to EMF could result in irreversible damage which may lead to subfertility and infertility. Hence it is suggested that long term exposure to EMF should be avoided and an opportunity to have an exposure free time be given. It is recommended that there should be exposure free interval.
